# The prognosis value of EphA3 and the androgen receptor in prostate cancer treated with radical prostatectomy

**DOI:** 10.1002/jcla.22871

**Published:** 2019-04-08

**Authors:** Xiuzhi Duan, Xiaoming Xu, Binbin Yin, Bong Hong, Weiwei Liu, Qian Liu, Zhihua Tao

**Affiliations:** ^1^ Department of Clinical Laboratory The Second Affiliated Hospital of Zhejiang University School of Medicine Hangzhou China; ^2^ Department of Pathology The Second Affiliated Hospital of Zhejiang University School of Medicine Hangzhou China; ^3^ Department of Clinical Laboratory Women’s Hospital School of Medicine Zhejiang University Hangzhou China; ^4^ International Medical Center Second Affiliated Hospital of Zhejiang University Hangzhou China

**Keywords:** androgen receptor, EphA3, overall survival, overexpression, prognosis, prostate cancer

## Abstract

**Background:**

This study aimed to preliminarily assess the relationship between erythropoietin‐producing hepatocellular carcinoma receptor A3 (EphA3) and androgen receptor (AR) protein expression levels and prognosis in prostate cancer (PCa) to better understand the role of EphA3 in the prognosis and progression of PCa.

**Materials:**

We investigated the expression of EphA3 and AR in human PCa by immunohistochemistry.

**Results:**

EphA3 and AR were both significantly upregulated in PCa, with expression mainly localized to the nucleus. A high level of AR expression was found in 48.4% of 64 tumor samples, which was significantly more than in the adjacent tissue samples (15.6%) (*P* < 0.01). The percentage of samples expressing a high level of EphA3 was significantly greater in the PCa samples (54.7%) than in the adjacent tissue samples (20.3%) for the 64 tumors (*P* < 0.01). The high levels of EphA3 and AR expression in the PCa tissue samples were both correlated with the pathological stage, bladder and rectal invasion, distant metastasis, and preoperative PSA level (both *P* < 0.05). The survival time was significantly shorter in high levels of AR expression of patients. (*P* < 0.01). A high level of EphA3 in PCa patients suggests a poor prognosis (*P* < 0.05). Biochemical recurrence, distant metastasis, and the final scores of EphA3 and AR expression were significantly correlated with the prognosis of PCa (*P* < 0.05).

**Conclusions:**

Increased EphA3 expression is an independent prognostic factor for a poor outcome and decreased survival in PCa.

## 1 INTRODUCTION

Prostate cancer (PCa) represents the highest proportion of new cancer cases and has the second highest mortality rate in males according to the latest cancer statistics by the American Cancer Society.^1^ Despite improvements in PCa diagnosis and multiple therapies, the 5‐year survival rate of patients with metastatic disease is only 29% in the United States.^2^ PCa also had the sixth highest incidence and mortality rate among the top 10 most common cancers in males in China in 2013, and PCa had the most obvious upward trend in incidence and mortality.^3^ The androgen receptor (AR), as a member of the nuclear receptor superfamily, plays an vital role in the male phenotype and PCa biology. PCa was originally identified as an androgen‐dependent tumor, and its growth and survival were found to be controlled by AR signaling.^4^ Androgen deprivation therapy is effective for inhibiting PCa growth by initially suppressing AR activity.^5^ However, this treatment is more likely to lead to recurrence of prostate cancer, and relapsed prostate cancer is not responsive to androgen deprivation therapy.^6^ Despite the loss of response to antiandrogens, data suggest that AR signaling continues to play a role in castration‐resistant prostate cancer.

The EphA3 gene is AR‐regulated and contains an AR genomic binding site that was confirmed by our previous research, and our previous study identified EphA3 as the first hypomethylated gene in a differentially methylated gene map. The mRNA level of EphA3 differs significantly (192‐fold) between androgen‐independent PCa and androgen‐dependent PCa cell lines according to a DNA methylation chip‐based study.^7^ Our previous data showing that the EphA3 gene is AR‐regulated and contains an AR genomic binding site was obtained using chromatin immunoprecipitation (ChIP) in combination with direct sequencing (ChIP‐seq). Singh et al demonstrated that EphA3 is significantly upregulated in castration‐resistant PCa cells, which exhibit differential expression during androgen‐independent progression.^8^ These results prompted the present study focusing on the correlation between EphA3 and AR in the malignant behavior of PCa.

Eph receptors (Ephs) are the largest family of receptor tyrosine kinases with fourteen receptors divided into two subfamilies (EphAs and EphBs); these receptors are associated with angiogenesis and tumor vasculature in various human cancers.^9^ EphA3 is highly expressed in the brain, kidneys, heart, and lungs during embryonic development and then declines to a low level in adults. However, EphA3 expression is also elevated in a wide range of malignancies, including hepatocellular carcinoma,^10^ glioblastoma,^11^ gastric cancer,^12^, ^13^ and melanoma,^14^ and is correlated with tumorigenicity, tumor angiogenesis, cancer progression, and poor prognosis.^10^, ^11^, ^12^, ^13^, ^14^ The EphA3‐specific monoclonal antibody IIIA4 was also found to have antitumor effects in EphA3‐expressing leukemic xenografts.^15^ KB004, a monoclonal antibody targeting EphA3, has been used in a multicenter phase I study of human hematologic malignancies.^16^ Accordingly, EphA3 has received more attention as a promising target for the treatment of several cancers.^17^ Conversely, there are also contradictory reports concerning EphA3 expression in tumors and its effect on the regulation of cancer progression. EphA3 expression is more commonly downregulated and does not play a major role in colorectal cancer.^18^ EphA3 has been found to suppress cell adhesion and migration when EphA3 phosphorylation is increased by ephrinA5 stimulation in EphA3‐expressing TE671 and RD rhabdomyosarcoma cells or when EphA3 is ectopically expressed in the EphA3‐negative CRL2061 rhabdomyosarcoma cell line.^19^ Overexpression of EphA3 promotes lung cancer cell apoptosis and inhibits tumor xenograft growth by inhibiting AKT activation.^20^ EphA3 is involved in regulating multidrug resistance via PI3K/BMX/STAT3 signaling and may be a new therapeutic target in small cell lung cancer.^20^ Overall, EphA3 plays a significant role in the progression of cancer, but the role of EphA3 in either promoting or suppressing oncogenesis in a variety of cancers is quite complex and paradoxical.

In the prostate, higher amounts of EphA3 have been reported in the normal prostate than in other benign human tissues.^21^ High expression of EphA3 in PCa may promote the development and progression of PCa.^22^ The mRNA expression level of EphA3 was higher in the androgen‐dependent PCa cell lines 22Rv1 and LNCaP with elevated AR expression levels than in the androgen‐independent PCa cell lines DU145 and PC‐3 without AR expression.^23^ Immunoprecipitation (IP) analysis of human tumor cell lines indicated the lack of EphA3 in DU145 cells, and androgen‐dependent PCa cell lines (LNCaP and 22Rv1 cells) were EphA3^+^.^23^ However, Brian et al demonstrated that the transcript level for EphA3 was elevated in LNCaP and PC‐3 cells but reduced in DU145 cells.^24^ We speculated that there might be some correlation between EphA3 and AR in PCa, which needs to be confirmed. In addition, as the Gleason score of prostate cancer increased in clinical PCa specimens, the expression of EphA3 increased and the proportion of nuclear localization also increased gradually by tissue microarray staining.^24^ Furthermore, EphA3 promoted the proliferation and survival of PCa LNCaP cells and tumor formation in nude mice subcutaneously by implanting with EphA3‐overexpressing LNCaP cells.^22^

Therefore, we attempted to preliminarily assess the relationship between EphA3 and AR protein expression levels in PCa and evaluated the prognostic impact of EphA3 to better understand the role of EphA3 in the progression and prognosis of PCa.

## 2 MATERIALS AND METHODS

### 2.1 Patient samples

Using a retrospective study design, PCa and adjacent benign tissues were obtained from 64 patients treated via radical prostatectomy between January 2010 and December 2017 at the Second Affiliated Hospital Zhejiang University School of Medicine (Hangzhou, China). The average age of the patients diagnosed with PCa was 73 (range 50‐85) years, and the mean follow‐up period was 67.62 (range 9‐94) months. This study was approved by the Second Affiliated Hospital Ethics Committee of Zhejiang University School of Medicine. All of the patients underwent radical prostatectomy and bilateral lymphadenectomy. None of the patients received hormone or radiation therapy before surgery, and none had secondary cancer. The Gleason score was assigned by two senior pathologists experienced in the diagnosis of PCa. Tumor staging was performed according to the American Joint Committee on Cancer classification system. Biochemical recurrence was defined as a PSA concentration of ≥0.2 ng/mL.

All of the stained sections were reviewed by two pathologists (XX, and BH). The follow‐up protocol at the institution included a clinical visit, physical examination, and contrast‐enhanced CT at day 7, month 3, month 6, and every 6 months thereafter.

### 2.2 Immunohistochemical analysis technique

The tissue sections were incubated with a mouse monoclonal antibody against EphA3 (Ab54623; Abcam, Cambridge, MA, USA) used at a 1:200 dilution, the AR mouse monoclonal antibody (441) (sc‐7305; Santa Cruz Biotechnology, Inc, Santa Cruz, CA, USA) used at a 1:100 dilution. Formalin‐fixed paraffin‐embedded tissues from PCa and the corresponding adjacent normal prostate tissues (at least 1.5 cm away from the tumor) were investigated from 64 patients. The EnVisionTM method (Dako, Glostrup, Denmark) was used for the immunohistochemical staining. Sections (5 μm) of a paraffin‐embedded tissue block were rehydrated by sequential immersion in graded ethanol solutions. Endogenous peroxidase activity was blocked with 3% hydrogen peroxide at room temperature for half an hour, and antigen retrieval was performed in citrate buffer at 100°C for 30 minutes. Then, the sections incubated in 10% normal goat serum for 5 minutes and with all monoclonal antibody anti‐EphA3 and AR antibody at 4°C overnight. Human gastric carcinoma tissue was used as a positive control sample for EphA3 expression and prostate tissue for AR expression. Phosphate‐buffered saline (PBS) was used instead of the primary antibody as the negative control.

### 2.3 Interpretation of the immunostaining results

The sections were viewed by two pathologists without knowledge of the clinical records of the patients, and any disagreements were resolved by reassessment and consensus. A previously developed semiquantitative system to determine EphA3 expression was used. A staining index (SI; values 0‐12) was calculated as a product of staining intensity (no staining = 0, light yellow/weak staining = 1, yellowish brown/moderate staining = 2, brown/strong staining = 3) and proportion of positive cells (0%‐5% = 0, 6%‐25% = 1, 26%‐50% = 2, 51%‐75% = 3;>75% = 4). Cutoff points for SI categories were based on median values.^25^ EphA3 was categorized by median values as high (SI ≥ 6) or low level (<6). AR was divided by its median values as high (SI ≥ 8) or low level (<8).

### 2.4 Statistical analysis

The statistical analysis was performed using SPSS (v20.0; IBM, Inc, Armonk, NY, USA) software. Significant differences were calculated using the chi‐squared test. Overall survival (OS) was used to evaluate the prognosis. The survival curves were obtained by the Kaplan‐Meier method and compared with the log‐rank test. The multivariate model used a Cox regression analysis. The statistical significance was set at *P* < 0.05 or 0.01 as indicated.

## 3 RESULTS

### 3.1 Clinical data

The median age at diagnosis for the 64 patients was 73 years (range, 50‐85). Of the 64 patients, 22 (34.4%) had a biochemical recurrence and 16 (25.0%) had distant metastasis (Table 1). The median OS for the 64 subjects was 81.70 months (95% CI, 75.45‐87.94 months). During the study period, 13 patients died of their cancer.

**Table 1:** Characteristics of patients with prostate cancer

Characteristic	PCa number (%)
Age	73 (50‐85)
Pathologic stage	
pT1‐2	44 (68.8)
pT3‐4	20 (31.2)
Gleason score	
≤7	33 (51.6)
>7	31 (48.4)
Biochemical recurrence	
Negative	42 (65.6)
Positive	22 (34.4)
Seminal vesicle invasion	
Negative	47 (73.4)
Positive	17 (26.6)
Bladder and rectal invasion	
Negative	55 (85.9)
Positive	9 (14.1)
Distant metastasis	
Negative	48 (75.0)
Positive	16 (25.0)
Initial PSA (ng/mL)	
≤10	31 (48.4)
>10	33 (51.6)

### 3.2 EphA3 and AR protein expression in human PCa

**Figure 1:**
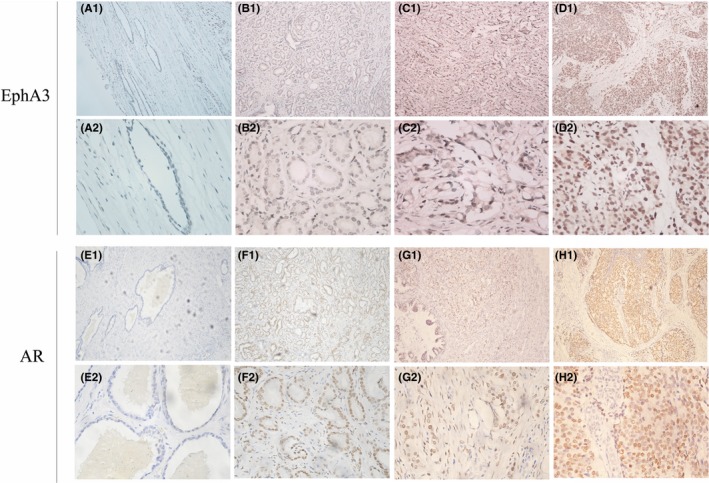
EphA3 and AR expression in prostate cancer tissue. (A, E) Adjacent prostate cancer tissue. (B, F) Gleason grade 3 of PCa. (C, G) Gleason grade 4. (D, H) Gleason grade 5. Original magnification ×100 (Row 1 and 3, A1‐D1 and E1‐H1), ×400 (Row 2 and 4, A2‐D2 and E2‐H2)

Positive staining of the AR protein was found in the nucleus, EphA3 was located in the nucleus and cytoplasm of the PCa tissues, and the adjacent normal prostate tissues all showed faint staining according to the IHC of successive tumor tissue sections (Figure 1). The two factors were both significantly upregulated in PCa with expression mainly in the nucleus, compared with their expression in the corresponding adjacent normal prostate tissues. The high level of AR expression was observed in 31 (48.4%) of 64 tumor samples, which was more than that in the adjacent tissue samples (10 [15.6%] of 64; *χ* ^2^ = 15.825, *P* = 0.000, chi‐squared test). While the occurrence of the high level of EphA3 expression in the PCa samples was greater (35 [54.7%] of 64) than that in the adjacent tissue samples (13 [20.3%] of 64; *χ* ^2^ = 16.133, *P* = 0.000, chi‐squared test; Table 2).

**Table 2:** Correlation between EphA3 expression and clinical characteristics of patients with PCa

Variables	N	AR (*P*‐value a)				EphA3 (P‐value a)			
		High	Low	% high	*P* (*χ* ^2^)	High	Low	%	*P* (*χ* ^2^)
*Tissues*									
a‐PC	64	10	54	15.6	0.000 (15.825)	13	51	20.3	0.000 (16.133)
PCa	64	31	33	48.4		35	29	54.7	
*Characteristic*									
Age (y)									
<73	28	14	14	50.0	0.825 (0.049)	16	12	57.1	0.570 (0.323)
≥73	36	17	19	47.2		18	18	50.0	
Pathologic stage									
pT1‐2	44	15	29	34.1	0.001* (16.603)	19	25	43.2	0.018* (5.590)
pT3‐4	20	16	4	80.0		15	5	75.0	
Gleason score									
≤7	33	11	22	33.3	0.013* (6.223)	14	19	42.4	0.077 (3.133)
>7	31	20	11	64.5		20	11	64.5	
Biochemical recurrence									
Negative	42	17	25	40.5	0.078 (3.101)	16	26	38.1	0.001* (11.803)
Positive	22	14	8	63.6		18	4	81.8	
Seminal vesicle invasion									
Negative	47	20	27	42.6	0.117 (2.435)	24	23	51.1	0.583 (0.302)
Positive	17	11	6	64.7		10	7	58.8	
Bladder & rectal invasion									
Negative	55	23	32	41.8	0.009* (6.861)	26	29	47.3	0.020* (5.379)
Positive	9	8	1	88.9		8	1	88.9	
Distant metastasis									
Negative	48	17	31	35.4	0.000* (13.034)	22	26	45.8	0.043* (4.099)
Positive	16	14	2	87.5		12	4	75.0	
Preoperative PSA									
≤10 (ng/mL)	31	11	20	35.5	0.044* (4.039)	9	22	29.0	0.000* (14.014)
>10 (ng/mL)	33	20	13	60.6		25	8	75.8	

a‐PCa, adjacent PCa tissues.

* Statistically significant (*P* < 0.05).

### 3.3 Association of clinicopathological parameters with EphA3 and AR expression

EphA3 and AR expression levels and their association with the clinical characteristics of the 64 PCa tissue samples are shown in Table 2. The high levels of EphA3 and AR expression in the PCa tissue samples were both correlated with the pathological stage, bladder and rectal invasion, distant metastasis, and preoperative PSA level (both *P* < 0.05). Neither correlated with age nor seminal vesicle invasion (both *P* > 0.05). Only high level of EphA3 was related to biochemical recurrence (*χ* ^2^ = 11.803, *P* = 0.001, chi‐squared test), and high AR expression was related to the Gleason score (*χ* ^2^ = 6.223, *P* = 0.013, chi‐squared test).

### 3.4 EphA3 and AR expression in the prognosis of PCa

**Figure 2:**
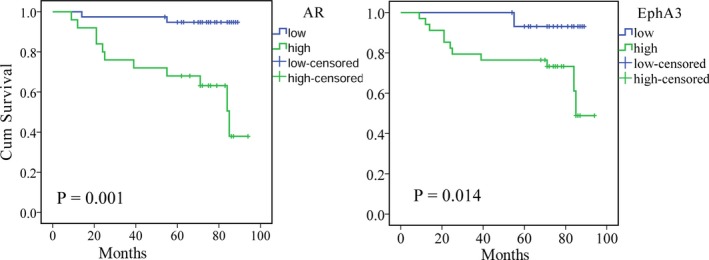
Kaplan‐Meier plots with log‐rank test of overall survival (OS). Kaplan‐Meier analysis showing that patients with pancreatic cancer with high EphA3 and AR expression had shorter survival than those with low EphA3 and AR expression

The Kaplan‐Meier survival curves of EphA3 and the AR are shown in Figure 2.

The survival time of patients with high level of AR expression was significantly shorter than that of patients with a low AR level (*P* = 0.001, log‐rank test). The high level of EphA3 in PCa patients suggests a poor prognosis (*P* = 0.014, log‐rank test). In the Cox regression analysis of OS (Table 3), biochemical recurrence, distant metastasis, and the final scores of EphA3 and AR expression were significantly correlated with the prognosis of PCa (*P* < 0.05). Moreover, the final scores of EphA3 expression were confirmed as an independent prognostic indicator of poor OS in multivariate analysis.

**Table 3:** Multivariate analysis of overall survival

Variable	HR (95% CI)	*P*‐value
Age	0.949 (0.201‐4.480)	0.947
Pathologic stage	0.349 (0.017‐6.990)	0.492
Gleason score	0.199 (0.018‐2.256)	0.193
Biochemical recurrence	0.042 (0.003‐0.690)	0.026*
Seminal vesicle invasion	8.148 (0.443‐149.8)	0.158
Bladder & rectal invasion	0.279 (0.020‐3.893)	0.342
Distant Metastasis	330.4 (6.863‐15913)	0.003*
Preoperative PSA	0.515 (0.102‐2.590)	0.421
EphA3 expression(score)	1.739 (1.013‐2.986)	0.045*
AR expression(score)	1.711 (1.022‐2.865)	0.041*

CI, confidence interval; HR, hazard ratio.

* Statistically significant (Cox regression analysis, *P* < 0.05).

### 3.5 Correlation of expression

The EphA3 and AR were positively correlated in PCa based on protein expression by analyzing the final scores of EphA3 and AR expression (*r* = 0.571, *P* = 0.001, Spearman correlation test).

## 4 DISCUSSION

Because of the limited prognostic data for EphA3 in PCa, we investigated for the first time the association between EphA3 and AR expression along with PCa prognosis using human PCa and adjacent tissues. Positive staining of the AR protein was found in the nucleus, and EphA3 was located in the nucleus and cytoplasm of the PCa tissues, and all adjacent normal prostate tissues showed faint staining. The same phenomenon regarding EphA3 expression was also reported by Wu et al,^22^ who showed using a human PCa tissue microarray that EphA3 is overexpressed in PCa specimens and that EphA3 is highly expressed in androgen‐independent and metastatic cell lines.

The relationship between EphA3 and AR expression and the clinical characteristics of PCa were further investigated in the present study. The results showed that both proteins were correlated with pathological stage, bladder and rectal invasion, distant metastasis, and the preoperative PSA level but were not correlated with age or seminal vesicle invasion. Biochemical recurrence was only related to high EphA3 expression levels, and the Gleason score was only related to a high level of AR expression. The high EphA3 level has also been correlated with histological grade, the invasion depth, distant metastasis and TNM stage in colorectal cancer^26^ and gastric cancer.^13^ A high expression level of EphA3 has been correlated with a high invasive capacity in hepatocellular cancer^10^ and mesenchymal glioblastoma.^11^ Regarding PCa, EphA3 is a significantly upregulated gene in androgen‐independent cells,^8^ and this phenomenon might be due to a mechanism of gene amplification or DNA methylation.^27^ In addition, Diao et al found that EphA3 expression was induced by the interaction of the AR with SP1 transcription factor.^23^ These findings suggest that there is some correlation between EphA3 and PCa progression.

A significant relationship between EphA3 expression and OS was observed. Using a hospital‐based retrospective analysis, the prognostic study showed that patients with high levels of EphA3 and AR expression had significantly shorter survival times. A high EphA3 level independent of other prognostic factors was associated with a lower OS rate. In multivariate analysis, the biochemical recurrence, distant metastasis, EphA3 and AR expression were significantly associated with the prognosis of PCa and were independent prognostic factors of poor OS. Although there have been few studies on EphA3 and PCa prognosis, in mesenchymal glioblastoma, hepatocellular carcinoma,^10^ gastric carcinoma,^12^ and multiple myeloma, it has been confirmed that high EphA3 expression is associated with a poor prognosis. These preliminary results indicate that EphA3 may be a potential oncogene that plays an important role in the malignant progression and prognosis of PCa.

Based on the previous description that the EphA3 gene may be an AR‐regulated gene, we analyzed the association between EphA3 and AR according to the Spearman correlation test using the sum staining scores of the immunohistochemistry analysis and found a positive correlation between these factors. Diao et al found that the EphA3 mRNA and protein levels were both elevated by the hormone DHT in a dose‐ and time‐dependent manner accompanied by increased AR expression. The overexpression of pEGFP‐AR in 22Rv1 cells significantly increased the EphA3 level, whereas AR knockdown with small interfering RNA (siRNA) specific for the AR (siAR) markedly decreased the expression of EphA3.^23^ Another study showed that prostate androgen induces the prostate leucine zipper gene‐promoted expression of EphA3.^22^ Overall, these results suggest that EphA3 expression is affected by the AR or possibly factors in the AR signaling pathway. The exact mechanism of this phenomenon is unclear and needs to be studied further.

## 5 CONCLUSIONS

Given the findings described above, EphA3 plays an important role both individually and together with other factors in the progression and prognosis of PCa. EphA3 may be a useful prognostic biomarker of PCa, with the presence of EphA3 suggesting a poor outcome and decreased survival. EphA3 affects the prognosis of PCa, potentially through the AR or a related pathway factor.

## ETHICAL APPROVAL

The study was approved by the Ethics Committee of The Second Affiliated Hospital, Zhejiang University School of Medicine. Informed consent was obtained from all the individual participants included in the study.
